# Accumulation and metabolism of selenium by yeast cells

**DOI:** 10.1007/s00253-015-6650-x

**Published:** 2015-05-24

**Authors:** Marek Kieliszek, Stanisław Błażejak, Iwona Gientka, Anna Bzducha-Wróbel

**Affiliations:** Department of Biotechnology, Microbiology and Food Evaluation, Faculty of Food Sciences, Warsaw University of Life Sciences − SGGW, Nowoursynowska 159 C, 02-776 Warsaw, Poland

**Keywords:** Selenium, Accumulation, Yeast, Cells

## Abstract

This paper examines the process of selenium bioaccumulation and selenium metabolism in yeast cells. Yeast cells can bind elements in ionic from the environment and permanently integrate them into their cellular structure. Up to now, *Saccharomyces cerevisiae*, *Candida utilis*, and *Yarrowia lipolytica* yeasts have been used primarily in biotechnological studies to evaluate binding of minerals. Yeast cells are able to bind selenium in the form of both organic and inorganic compounds. The process of bioaccumulation of selenium by microorganisms occurs through two mechanisms: extracellular binding by ligands of membrane assembly and intracellular accumulation associated with the transport of ions across the cytoplasmic membrane into the cell interior. During intracellular metabolism of selenium, oxidation, reduction, methylation, and selenoprotein synthesis processes are involved, as exemplified by detoxification processes that allow yeasts to survive under culture conditions involving the elevated selenium concentrations which were observed. Selenium yeasts represent probably the best absorbed form of this element. In turn, in terms of wide application, the inclusion of yeast with accumulated selenium may aid in lessening selenium deficiency in a diet.

## Introduction

Selenium is a member of a group of trace elements, which are essential to proper functioning of an organism. It is an integral part of selenoproteins and several antioxidant enzymes such as glutathione peroxidase (GPx), thioredoxin reductase (TRxR), and iodothyronine deiodinase (DIO), which protect cells from the harmful effects of free radicals that are generated during the oxidation process (Drutel et al. 2013). Due to rapid metabolism, yeast cells are characterized by a high level of interaction with the extracellular environment. A characteristic feature of fodder yeasts is its rapid proliferation, which is described by the intense increase of cell biomass. In consequence, efficient assimilation and conversion of many minerals from the environment are observed in the cellular structure of yeasts. Many trace elements, including those essential for life, such as selenium, are naturally accumulated by yeast (Kieliszek and Błażejak 2013; Schrauzer 2006). Yeast cells are widely used in the production of food and fodder as well as in the biotechnology and pharmaceutical industries. In addition, they can be employed as a eukaryotic cell culture model in research (Brozmanová et al. 2010). It is worth emphasizing that the mechanism of accumulation of selenium and its conversion into cellular structures is not fully understood. This paper attempts to describe the possible processes of accumulation and transformation of the element selenium in yeast cells.

## Mechanisms of external binding of selenium

Extracellular binding of selenium is based on chemisorption. This process involves the formation of ionic bonds or complexation of selenium ions by biopolymers of the yeast cell wall, such as active groups of proteins, phospholipids, or polysaccharides.

The cell wall of yeasts represents 10 to 30 % of the dry weight of their cell biomass and is mostly composed of polysaccharides (85 %) and proteins (15 %). Chemical analyses of the polysaccharide show that it includes glucose (80–90 %), mannose (10–20 %), and N-acetylglocosamine (1–2 %) (Klis et al. 2006). The structure of the cell wall consists of two layers, an outer layer of mannoproteins (30–50 % of the cell wall) and an inner layer of carbohydrate polymer β-(1,3) glucan (30–45 %) (Saluk-Juszczak et al. 2010). The cell wall is also composed of β-(1,6) glucan (8–18 %) and chitin amounting to between 1 and 2 % of the total composition of the cell wall (Levin and Moran 2011; Orlean 2012).

Mannoproteins, commonly known as proteoglycans (Lipke and Ovalle 1998), are highly glycosylated polypeptides rich in mannose (carbohydrates constitute 50–95 % of their molecular weight). This reduced permeability is caused by the presence of long branched carbohydrate chains linked to the polypeptide chain through O-glycoside bonds with hydroxyl groups of serine or threonine. N-glycosidic bonds can also be distinguished to be linked to the amide group of asparagine, which in consequence causes the formation of a rigid structure of densely packed polypeptide regions constituting a “scaffold” for the cell wall (Klis et al. 2006; Orleans 2012).

All components of the cell wall are interconnected via covalent bonds to form a homogeneous structure. Low molecular weight but highly branched and water-soluble β-(1,6) glucan is covalently bound to β-(1,3) glucan, which is branched only to a small extent. Additionally, β-(1,6) glucan is linked to chitin via β-(1,4) glycosidic bonds (Bzducha-Wróbel et al. 2013; Hurtado-Guerrero et al. 2009; Orleans 2012).

The entire complex is linked by mannoproteins through glycosylphosphatidylinositol (GPI) anchor in the outer layer of the cell membrane (Lesage and Bussey 2006; Jigami and Odani 1999). Such a system forms an organized and highly flexible structure (Levin and Moran 2011), affecting the stabilization of the cell wall (Klis et al. 2006; 2002).

Physicochemical processes play a major role in the extracellular binding of selenium. The mannoprotein layer which forms an outer protective barrier and which determines the permeability of the cell wall of yeast is of particular importance (Caridi 2006). Biosorption of selenium occurs due to the presence of functional groups that exhibit a negative charge on the surface of the cell wall; for example, phosphodiester (Klis et al. 2002) and sulfide (Lipke and Ovalle 1998) bridges, mannose phosphate residues (Caridi 2006; Kordialik-Bogacka 2011), and negatively charged phosphate, and carboxyl and hydroxyl groups (Tobin et al. 1994). The extent of biosorption is strongly influenced by the hydrophobicity of the surface of the yeast cell wall, which is dependent on the presence of polysaccharides, proteins, and lipids (Kordialik-Bogacka 2011).

In order to determine the role of the cell wall in the process of bioaccumulation of selenium, Chmielowski et al. (1991) studied a cell culture of *Saccharomyces cerevisiae* SBTD. The study involved whole cells and protoplasts. To identify selenium in the yeast cell wall, the authors used the addition of sodium selenite (IV), Se^75^ isotope. It was observed that whole yeast cells cultured in a medium containing glucose bound selenium in an amount of 5.5 mg/g, while in a medium containing fructose, the cells bound 7.9 mg/g. Cells from which the cell wall had been removed enzymatically took up 4.4 and 6.3 mg/g from glucose and fructose medium, respectively.

In protoplasts, the selenium content of protoplasts was observed to have decreased by 20 %, which confirmed the important role of cell wall polysaccharides in the studied process. At the same time, intracellular accumulation of selenium accelerated. Based on this information, it can be assumed that the cell wall and its polysaccharide components constitute a barrier reducing the penetration of selenium into the cell interior. In the available literature, there is no information on the importance of cell wall proteins in the process of selenium binding.

Based on numerous studies evaluating the accumulation of selenium in yeast cells and in cell wall polysaccharides, Chmielowski and Tyflewska (2007) have reported that mannan exhibits a much greater ability of selenium sorption in comparison to glucan and chitin. It has been shown that the amount of selenium bound by polysaccharides increased with increasing concentrations of selenium in the culture medium. Based on the obtained results, it was found that a chemisorption process was responsible for the binding of selenium by mannan and glucan.

Studies on the usefulness of bacteria in terms of binding of selenium from aqueous solutions were published in 1997 by Losi and Frankenberger (1997). One study showed that *Enterobacter cloacae* SLD1a-1 was able to reduce selenium (VI) (selenate), which was bound to its cell wall, to elemental selenium. The whole process occurred near the cell plasma membrane. According to the biochemical studies presented by Yee et al. (2007), selenate reductase closely bound to the cytoplasmic membrane is responsible for the reduction of selenium. The obtained results demonstrate that bacteria may be used in bioremediating selenate-contaminated soils, sediments, and industrial wastes (Ridley et al. 2006; Sarret et al. 2005).

Čertík et al. (2013) demonstrated that the addition of selenium to the culture medium caused a change in the fatty acid profile of the cytoplasmic membrane of yeast; they observed an increase in the content of C-18 fatty acids. On the other hand, in yeast enriched in carotenoids, they noticed a decomposition of unsaturated fatty acids (linoleic and linolenic acids).

Using electron microscopy techniques, the authors of other publications (Gerrard et al. 1974) demonstrated that *Escherichia coli* cultured in medium containing 340 mg selenite per liter was able to bind the selenium in the outer membrane and in the cytoplasmic membrane of bacteria, as well as in between them. It showed that the defense mechanism involved the accumulation of selenium in the cell membranes, thereby preventing the penetration of selenite ions into the cytosol.

## Intracellular accumulation of selenium

The process of intracellular accumulation of selenium occurs through active transport inside the cell interior of yeasts. To overcome the impermeability of the cell membrane to selenium ions, a specific transport mechanism is required. So far, only a few reports describing this process in yeast cells have been published. Furthermore, there are no studies on identification of selenium carriers at the molecular level (Rosen and Liu 2009). Analysis of the available research (Gharieb and Gadd 2004; Ponce de León et al. 2002) indicates that the majority of publications devoted to the study of intracellular accumulation of selenium relates mainly to yeast and bacteria.

Studies conducted by Sirko et al. (1990) and Turner et al. (1998) demonstrated that selenium is absorbed by *Escherichia coli* through sulfur ABC membrane transporters which are encoded by *cysAWTP* operon (Shaw et al. 2012). The transport complex is composed of two CysA molecules which bind ATP nucleotides, two integral membrane proteins (CysT and CysW), and CysP periplasmic sulfate-binding protein (Rosen and Liu 2009). This is an example of active transport, which occurs with the contribution of specialized, integral proteins.

ABC pumps for the transport of selenium ions use the energy derived from hydrolysis of bound ATP. A distinct selenium transport system based on sulphate permease has also been reported. This correlation was confirmed by Cherest et al. (1997) in experiments involving *S. cerevisiae*, wherein Sul1p and Sul2p specific transport systems exhibiting a high affinity toward sulfur were used to transport *SeO*_3_^2 −^ ons. In contrast, Zhil’tsova et al (1996) found that *Candida utilis* VSB-651 with high activity of reductase enzymes and glutathione peroxidase was able to bind two times more selenium compared to *Candida ethanolica* VSB-814 with low activity of these enzymes.

Danch and Chmielowski (1985) studied the binding process of selenium (IV) by *S. cerevisiae* D37. They found that the content of selenium in yeast cells grown in the presence of glucose and fructose reached the level of selenium equal to 14 and 11 mg/g, respectively. They also observed that the content of selenium in the nuclear fraction isolated from the cells after cultivation enriched with fructose was significantly higher (86 %) as compared to the fraction isolated from cells cultured in medium containing glucose (59 %).

Depending on the type of sugar in the culture medium, the increased content of selenium in the nuclear fraction and the total selenium accumulation denoted the presence of different transport mechanisms. On this basis, it was assumed that complexed selenium ions with an absorbed sugar substrate were transported to the cell cytosol. Kinetics of intracellular binding of selenium in *S. cerevisiae* indicated the existence of two transport systems—one with high and the other with low affinity toward selenium. Both types of transports were dependent on the presence of glucose in the culture medium, which significantly increased the rate of sorption of selenium by the yeast cells (Rosen and Liu 2009; Gharieb and Gadd 2004).

Falcone and Nickerson (1963) observed with *Candida albicans* 806 a similar correlation between the selenium content of the culture medium and the assimilation of glucose. The authors reported that sodium selenite (IV) inhibited the anaerobic process of assimilation of glucose by yeast. The use of oxygen in the medium was reduced by 84 %, while the carbon dioxide emission during fermentation decreased by only 63 %. This phenomenon was explained by the occurrence of strong inhibition of glucose oxidation by sodium selenite (IV). Simultaneously, for *Candida albicans* and *S. cerevisiae*, it was demonstrated that phosphate ions compete with selenite (IV) ions for binding sites in transport systems. An inhibitory effect of 2.4-dinitrophenol (DNP) was also observed (Falcone and Nickerson 1963).

Studies presented by Gharieb and Gadd (2004) and Lazard et al. (2010) showed that the presence of phosphate (V) and sulphate (IV) ions in the culture medium had an inhibitory effect on the binding affinity of selenium (IV) by *S. cerevisiae* yeasts. It was also reported that in the presence of sulphate (IV) ions, the yeast cells had the ability to carry out the reduction of selenite (IV) ions to elemental selenium. In addition, the presence of metabolic inhibitors, 2,4-dinitrophenol, potassium cyanide, sodium azide, and N-ethylmaleimide, influenced the reduction of selenium biosorption by yeast cells.

The presence of different substances in the culture medium has led to the theoretical considerations on the accumulation of selenium by yeast. Studies conducted by Gharieb and Gadd (2004) have shown that selenium transport was inhibited by the presence of sulfur in the molecules of exogenous amino acids like methionine, cysteine, and cystine. The presence of high concentrations of sulfate (IV) and (VI) in the culture medium did not affect the biosorption of selenium by *S. cerevisiae*.

Observations by other authors (Golubev and Golubev 2002) confirmed that the tolerance of yeast to the presence of selenium in the culture medium depends on the medium composition and the presence of sulfur amino acids. According to Demirci and Pometto (1999), a Se/S ratio estimated at 4:1 in the culture medium is optimal for efficient bioaccumulation of inorganic selenium and its transformation into organic forms by yeast cell biomass. Thus, the authors showed a correlation between selenium binding and the occurrence of metabolic products of sulfur (Gharieb and Gadd 2004).

The studies conducted by Lazard et al. (2010) showed that accumulation of selenite ions by *S. cerevisiae* was determined by the presence of phosphate ions in the culture medium. Based on this experiment, it was found that Pho84p and Pho89p transporters were major factors contributing to the binding of selenite (IV) ions by yeast in a culture medium with low phosphate content. At higher concentrations of phosphate in the medium, selenium transport was gradually replaced by low-affinity transporters (Pho87p, Pho90p, and Pho91p). As a consequence, absorption of selenium was reduced, and cell resistance toward increased doses of selenium increased.

Studies published by McDermott et al. (2010) demonstrated that symporter Jen1p monocarboxylic conveyor is responsible for the transport of selenite (IV) into the yeast cell interior. It is involved in the transport of pyruvic acid, lactic acid, acetic acid, and propionic acid (Paiva et al. 2013). Transport of selenite (IV) is based on the structural similarity of selenium anions and the anions of carboxylic acids. In addition, these molecules have similar dissociation constants, while within physiological pH, they are mono-negative anions. Under anaerobic environment in the absence of fermentable substrates, increased accumulation of selenium via Jen1p conveyor was observed. This phenomenon was explained by the use of carboxylic acids in the absence of preferred carbon sources, e.g., glucose.

Suhajda et al. (2000) conducted experiments in which the influence of culture conditions on the bioavailability of selenium to *S. cerevisiae* was evaluated. Based on the obtained results, it was found that the active acidity of the culture environment and the level of dissolved oxygen in the medium were the most important influence factors. In addition, the dynamics of biosorption of selenium by yeasts were influenced by the concentration and the type of this element (Pérez-Corona et al. 2011).

Selenium in an organic form exhibits improved properties in terms of biosorption by yeast cells and is less toxic than its inorganic form (Zhan et al. 2011). It should be stressed that binding of selenium by yeasts decreases with high contents of sulfur and heavy metals in the culture media. Furthermore, the presence of glucose may cause a reduction of selenium occurring in the form of *SeO*_3_^2 −^ ions, which results in the formation of red elemental selenium (Mapelli et al. 2011).

Different observations were made by Marinescu et al. (2011) who found that the process of selenium bioaccumulation by yeast cells was occurring mainly in the logarithmic growth phase. *Saccharomyces uvarum*, the brewer’s yeast, cultured in medium containing molasses wort supplemented with sodium selenite (IV), at concentrations from 30 to 180 mg/L, accumulated a large amount of selenium from 625.81 to 2215.67 μg/g.

Gharieb et al. (1995) studied the process of selenite (IV) reduction in filamentous fungi and yeasts cultured using different media containing selenium salt (Na_2_SeO_3_) as a selenium source at a concentration from 170 to 1700 mg/L. They found that depending on the presence of nutrients in the culture media, fungi under the study exhibited the possibility to perform the process of reduction of inorganic selenium (IV) to its elemental form (Se^0^). In terms of *Fusarium* sp. and *Trichoderma reesi* molds, selenium reduction proceeded using Czapek-Dox Agar medium resulted in the occurrence of the red color of the colony. *Aspergillus niger*, *Mucor* SK, and *Rhizopus arrhizus* fungi reduced the selenium to its elemental form (Se^0^) only on the malt extract agar (MEA). The occurrence of pink-red color of microbial biomass indicated on the accumulation of elemental selenium in the cell structures.

The study made by Ruocco et al. (2014) showed that *Rhodotorula mucilaginosa*-13B yeasts have the ability to reduce selenium in its structures. Microscopic observations have confirmed the presence of elemental selenium deposited in yeast cells. The above mentioned processes may be used in bioremediation processes of the selenate ion from waters contaminated with selenium.

Ponce de León et al. (2002) conducted studies in which different methods of selenium dosage were used for the experimental culture of *S. cerevisiae*. They showed that the best method to obtain selenium yeasts rich in one of the most favorable organic forms of this element (L-selenomethionine) was to add lower doses of sodium selenite (IV) (from 10 to 50 mg/L) in the early logarithmic growth phase of yeasts (Gharieb and Gadd 2004). The highest content of selenium in yeast cells (2354 μg/g) was obtained in the experimental medium supplemented with sodium selenite (IV) at a dose of 50 mg/L (Ponce de León et al. 2002). Yin et al. (2010) showed that optimal parameters for the enrichment of *S. cerevisiae* with selenium were temperature of 27.4 °C and active acidity (pH) at the level of 5.8.

The process of absorption of selenium by *S. uvarum* was described by Marinescu et al. (2011). They found that yeast cultured for 24 h in malt wort with sodium selenite (IV) at a concentration between 30 and 180 μg/mL bound large amounts of selenium (625–2215 μg/g). When wastewater from the brewing industry was used as a culture medium, it was observed that yeasts bound much less selenium (412–1624 μg/g). The authors noted that a significant increase in the accumulation of selenium by yeast biomass was brought about by temperature (30 °C) and by the addition of selenium to the culture medium during the initial phase of yeast growth.

Demirci and Pometto (1999) reported that the use of sodium selenite (VI) at a dose of 280 mg/L in continuous culture of *S. cerevisiae* caused a reduction in biomass productivity to 0.7 g/L. The content of selenium in yeast cell biomass was 687 μg/g. Comparing to the culture in which sodium selenite (IV) (690 mg/L) was used, one reported an increase in the content of selenium on biomass of yeast cells (1904 μg/g) was noticed. In addition, the production efficiency of yeast cell biomass was 1.8 g/L.

In conclusion, it should be stressed that binding of selenium by microbial cells largely depends on the culture conditions, the concentration of selenium in experimental medium, and the organisms used. It has an impact on the yield of the biomass and the content of selenium in the cell biomass. The mechanism of transport and bioaccumulation of selenium is associated with the presence of different transport conveyors or the existence of nonspecific transport of ions complexed with sugar substrates (Chmielowski and Tyflewska 2007).

## Metabolism of selenium by yeast cells

**Fig. 1:**
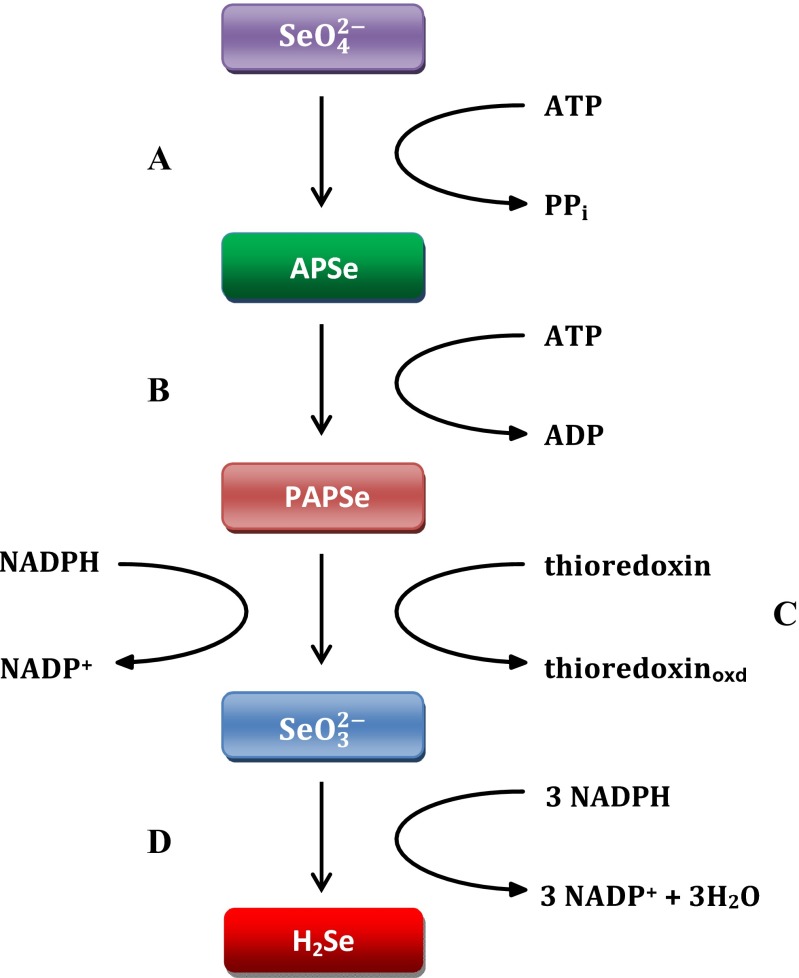
Pathway of reduction of selenium in yeast cells (Hoefig et al. 2011; Kitajima and Chiba 2013; Mapelli et al. 2011). **a** Sulfurylase ATP; **b** kinase APSe; **c** reductase PAPSe; **d** sulphate reductase

Because of the chemical similarity of such elements as sulfur and selenium, microorganisms absorb selenium inside the cell interior most likely by using enzymatic conveyors, i.e., Sul1 and Sul2 sulfate permeases (Mapelli et al. 2012). This suggests that transport of selenate (VI) is strictly dependent on the presence of sulfate (VI) in the culture medium. Under conditions of sulfur deficiency, the ability of selenium absorption by microorganisms increases. Studies of other transport systems in which phosphate and monocarboxylic acid transport is involved and which may play a role in selenium absorption have also been conducted (McDermott et al. 2010; Lazard et al. 2011). An enzymatic reduction is observed in the first stage of selenium metabolism in yeast cells (Fig. 1).

Initially, selenate (VI) is converted in an enzymatic process to APSe in a reaction catalyzed by ATP sulfurylase. The product formed in the reaction catalyzed by PAPSe reductase is converted into selenite (IV). In the described reaction, NADPH is a reducing agent (Bánszky et al. 2003). In the first route, selenite (IV) reduction is catalyzed by sulfate reductase using NADPH as reducing agent.

**Fig. 2:**
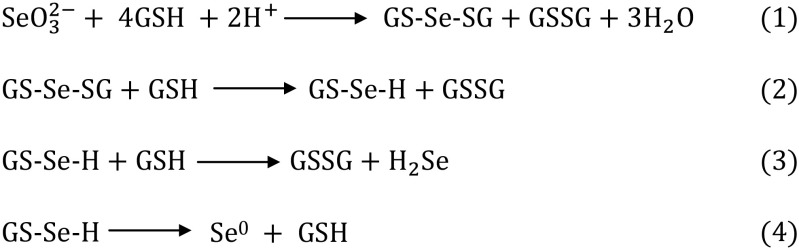
Transformation *SeO*_3_^2 −^ with the participation of glutatione

The second transformation process of *SeO*_3_^2 −^ with the participation of glutathione (Fig. 2) is slightly more complicated because selenite (IV) reacts spontaneously with the reduced form of glutathione (GSH). As a result, selenodiglutathione (GS-Se-SG) and the oxidized form of glutathione (GSSG) (reaction 1) are formed. The oxidized form of GSSG as a hazardous compound (forms disulfides with thiol-containing proteins and oxidizes them) is transported to the vacuole or converted into a reduced form (GSH) by glutathione reductase. In a further step, intracellular selenodiglutathione is converted into glutathionyselenol (GS-Se-H), and then to hydrogen selenide (H_2_Se/HSe^−^), with simultaneous formation of the oxidized form of glutathione (glutathione disulfide, GSSG) (reactions 2 and 3).

Volatile compounds of hydrogen selenide can freely pass through the vacuolar membrane into the cytoplasm of the cell via passive transport, according to concentration gradient. As a result of such series of reactions, transport of glutathione disulfide to a vacuole without the accumulation of selenium is observed (Lazard et al. 2011; Mapelli et al. 2011; Tarze et al. 2007). Glutathionylselenol can undergo further transformations forming elemental selenium and glutathione in the presence of superoxide dismutase (reaction 4) (Tarze et al. 2007).

According to Tarze et al. (2007), hydrogen selenide (H_2_Se) has the ability to penetrate into the cell via a passive way. This compound is formed through an effect of selenite (IV) and reducing agents. These reducing agents involve sulfhydryl groups that are present in the cell wall or cell membrane of yeasts. Other compounds exhibiting high efficiency of activity as reducing agents are glutathione and cysteine molecules. It is highly probable that the increased intake of selenium in the form of H_2_Se from the environment results from the presence of glutathione molecules in the cell or secreted into the extracellular space by yeasts.

**Fig. 3:**
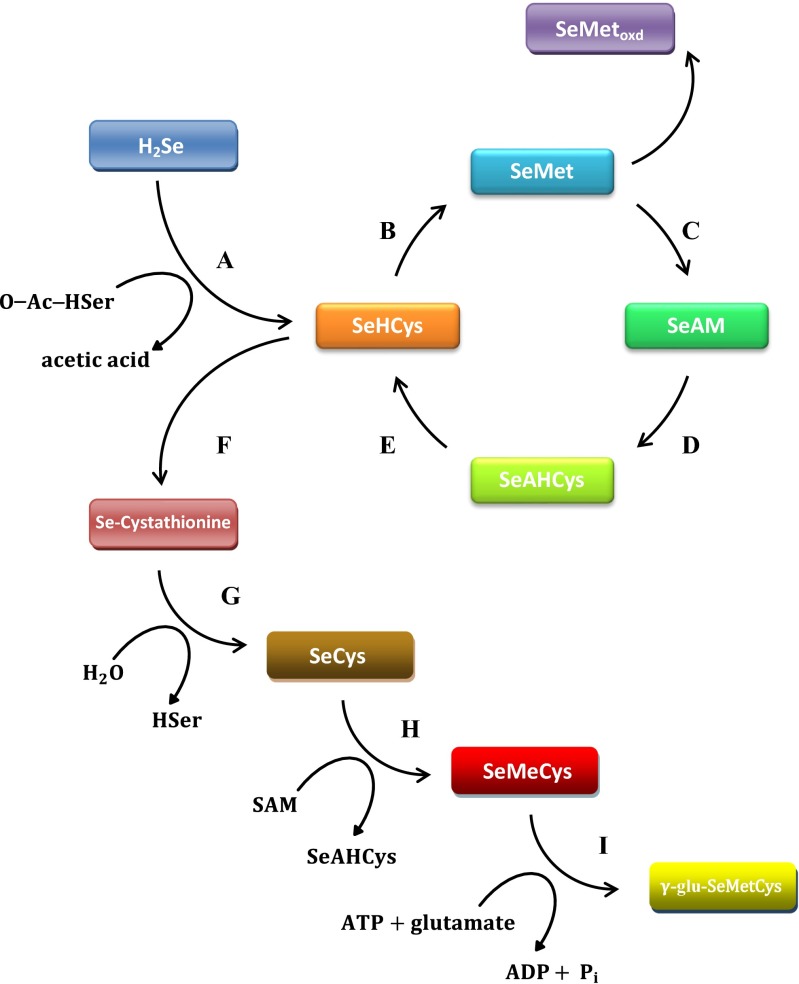
Schematic overview of the metabolism of the different selenocompounds in yeast (Hoefig et al. 2011; Kitajima and Chiba 2013; Mapelli et al. 2011). **a** Homocysteine synthase; **b** methionine synthase; **c** synthase adenosylomethionine; **d** methyltransferase; **e** hydrolase adenosylhomocysteine; **f** cystathionine-β-synthase; **g** cystathionine-γ-lyase; **h** Se-methyltransferase; **i** synthase γ-glu-Cys

The resulting hydrogen selenide is the major intermediate metabolite involved in the synthesis pathway of all forms of selenium occurring in microbial cells (Fig. 3). It is further metabolized forming organic compounds, including seleno amino acids. The first step of the reaction is the biosynthesis of homoselenocysteine. Hydrogen selenide is bound to O-acetylhomoserine (O-Ac-HSer) involving homocysteine synthase, and as a consequence, the formation of selenohomocysteine (SeHCys) and acetic acid is observed.

In subsequent steps, selenohomocysteine may be converted into selenocystathionine or selenomethionine (SeMet). In the first case, selenohomocysteine is bound to serine involving cystathionine β-synthase forming selenocystathionine and water. In the second case, homoselenocysteine is subjected to a methylation process, resulting in the formation of selenomethionine. The reaction is catalyzed by homocysteine methyltransferase (Kitajima and Chiba 2013; Mapelli et al. 2011).

The resulting selenomethionine, in the presence of oxygen, can be converted to its oxidized form (Pedrero et al. 2007; Schrauzer 2006). In the reaction catalyzed by S-adenozylomethionine synthase, selenomethionine (SeMet) is converted to Se-adenosyl-selenomethionine (SeAM). In the subsequent reaction, SeAM is subjected to enzymatic methylation process; as a result of which, adenosyl homo-seleno cysteine (SeAHCys) is released (Arnaudguilhem et al. 2012). Subsequently, the resulting compound undergoes hydrolysis, which results in the formation of selenohomocysteine (SeHCys) (Kitajima and Chiba 2013).

**Fig. 4:**
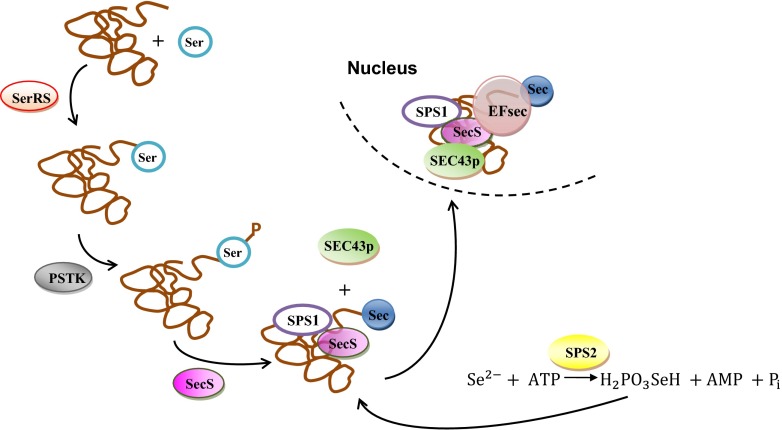
Selenocysteine biosynthesis in yeast cells (Allmang et al. 2009; Squires and Berry 2008; Turanov et al. 2011; Xu et al. 2007)

Selenocysteine (SeCys) is formed as a result of the transformation of selenomethionine using cystathionine γ-lyase enzyme. In subsequent reactions, selenocysteine reacts with S-adenosylmethionine (SAM) and may be converted into seleno-methyl-selenocysteine (SeMeCys) and S-adenosil-homo-selenocysteine by selenomethyltransferase (SMT). In the next step of the described transformations, Se-methylselenocysteine is converted into γ-glutamyl-Se-methyl cysteine (Mapelli et al. 2011). The next stage of the transformation of selenium in yeast cells is the incorporation of selenocysteine into proteins (Fig. 4). The incorporation process is possible through a specific Sec-tRNA^Sec^ complex.

During the first stage, tRNA^Sec^ is an aminoacylated serine, which provides the carbon skeleton for selenocysteine, and thus, Ser-tRNA^Sec^ is formed (Xu et al. 2005). The reaction requires the energy input supplied by ATP and is catalyzed with the contribution of conventional seryl-tRNA synthetase (SerRS). Next, the entire complex is phosphorylated by O-phosphoseryl-tRNASec kinase (PSTK). The next step is conversion of Ser-tRNA^Sec^ into Sec-tRNA^Sec^ using monoselenophosphate (H_2_PO_3_SeH) as a donor of activated selenium.

The resulting Sec-tRNA^Sec^ complex is transported by Secp43 factor to the nucleus, where incorporation of selenocysteine into proteins occurs (Allmang et al. 2009; Turanov et al. 2011). Selenocysteine is encoded by a specific UGA codon (Rayman 2004), which also constitutes as a terminal codon (Papp et al. 2007). During the synthesis of the polypeptide chain, the translational complex recognizes UGA codon thanks to the interaction of *trans* elements—SBP2-binding protein, acting as an elongation factor of EFSec protein, and cis element that is a specific messenger RNA (mRNA) secondary structure of a characteristic nucleotide sequence known as SECIS.

This sequence is located within a non-coding sequence at 3′-end of mRNA (3′UTR) (Bubenik et al. 2013; Fagegaltier et al. 2000; Suzuki et al. 2005). In a further stage, binding of EFSec-Sec-tRNA^Sec^ to ribosomal initiation complex via interaction with SBP2-binding protein, L30, and the SECIS structure is observed (Papp et al. 2007). Secp43 is responsible for the formation and stabilization of the entire protein complex (Bifano et al. 2013; Papp et al. 2007; Squires and Berry 2008).

**Fig. 5:**
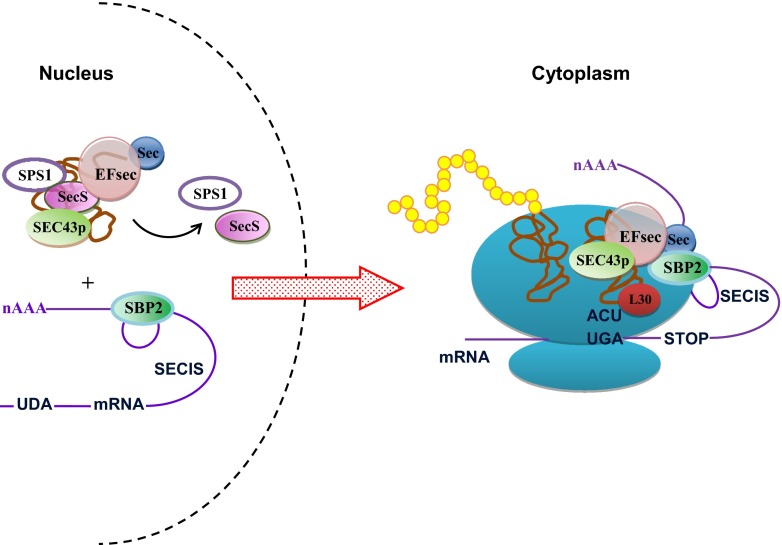
Selenoprotein biosynthesis in yeast cells (Allmang et al. 2009; Small-Howard et al. 2006; Squires and Berry 2008)

The translation complex, formed while reading the information contained in mRNA, reads the nucleotide sequence and then translates it into an amino acid sequence (Fig. 5). After reading UGA codon, Sec-tRNA^Sec^ is directed to the acceptor site on a ribosome where process of synthesizing selenium proteins occurs (Driscoll and Copeland 2003).

Enrichment of yeast with selenium is a consequence of the formation of many different selenium proteins. Selenomethionine is the basic form of selenium in yeast cells. Many authors, in their publications on selenomethionine determination (Gharieb and Gadd 2004; Rayman 2012; Schrauzer 2000; Tapiero et al. 2003), emphasize that it may constitute up to 90 % of the total content of selenium in yeast cells. Selenomethionine is the most absorbable form of selenium in human and animal organisms. It exhibits antioxidant properties, improves the immunity of an organism, and stimulates the activity of DNA repair enzymes (Laffon et al. 2010).

Selenomethionine is nonspecifically incorporated into proteins instead of methionine (Dumont et al. 2006; Letavayová et al. 2006). Moreover, it is effectively stored in tissues (Rayman 2008; Tapiero et al. 2003). Among other selenium compounds found in yeast cells, one can include selenocysteine, selenocystationine, Se-methylselenocysteine, and γ-glutamyl-Se-methylselenocysteine (Schrauzer 2000). During research on the identification of selenium proteins occurring in yeast cell biomass, more and more attention is being paid to anticancer compounds, e.g., Se-methylselenocysteine.

According to Kitajima and Chiba (2013), metabolites associated with the amino acids biosynthesis in yeast exhibit rather low toxicity. Interference with cell proliferation via Se-adenosylmethionine capable of DNA methylation is mainly considered in the context of metabolite action. However, it was also found that hydrogen selenide and reactive oxygen species generated by its presence could exert harmful effects. High concentration of these substances results in a strong redox imbalance and oxidation of important cellular elements such as proteins or DNA. According to El-Bayoumy et al. (2012), specific changes occur also in the proteome of selenium-enriched yeasts. The increased expression of proteins, such as HSP70, pyruvate kinase, and elongation factor 2, is observed.

In comparison to the other forms of selenium occurring in yeast cells, Se-methylselenocysteine exhibits strong anticancer properties. In humans and animals, Se-methylselenocysteine is converted into a compound methylselenol (CH_3_SeH) characterized by the highest anticancer activity. Another example of an organic selenium compound occurring in yeast is selenocysteine. It is widely considered as the 21st natural amino acid (Bubenik et al. 2013; Rayman 2008). It is involved in the biosynthesis of selenium proteins.

## Conclusion

Metabolism of selenium in yeast cells is a very complex process. A careful analysis of the forms and transformations which selenium compounds are subjected to in yeast will allow for a better understanding of its bioaccumulation and speciation. The metabolism of selenium compounds in cells is based on a series of transformations leading to a reduction of the degree of oxidation followed by the formation of selenide (H_2_Se). This is a common intermediate metabolite and, depending on the demand, may be used for the synthesis of selenoproteins or may be converted into methylated forms followed by their elimination from the organism in the same form. Another possibility is the formation of elemental selenium in yeast cell structures.

There is also the possibility of using selenium-enriched yeast biomass to produce protein and mineral preparations to be used as supplements to overcome deficiencies of this element in the diet.
